# Implementing shared decision-making on acute psychiatric wards: a cluster-randomized trial with inpatients suffering from schizophrenia (SDM-PLUS)

**DOI:** 10.1017/S2045796020000505

**Published:** 2020-06-16

**Authors:** J. Hamann, F. Holzhüter, S. Blakaj, S. Becher, B. Haller, M. Landgrebe, M. Schmauß, S. Heres

**Affiliations:** 1 Klinik und Poliklinik für Psychiatrie und Psychotherapie, Technische Universität München, Germany; 2 Isar-Amper-Klinikum München Ost, Haar, Germany; 3 Institut für Medizinische Informatik, Statistik und Epidemiologie, Technische Universität München, Germany; 4 Lech Mangfall Klinikum, Agatharied, Germany; 5 Bezirkskrankenhaus Augsburg, Germany

**Keywords:** Health service research, inpatient psychiatry, other psychosocial techniques/treatments, schizophrenia

## Abstract

**Aims:**

Although shared decision-making (SDM) has the potential to improve health outcomes, psychiatrists often exclude patients with more severe mental illnesses or more acute conditions from participation in treatment decisions. This study examines whether SDM is facilitated by an approach which is specifically adapted to the needs of acutely ill patients (SDM-PLUS).

**Methods:**

The study is a multi-centre, cluster-randomised, non-blinded, controlled trial of SDM-PLUS in 12 acute psychiatric wards of five psychiatric hospitals addressing inpatients with schizophrenia or schizoaffective disorder. All patients fulfilling the inclusion criteria were consecutively recruited for the trial at the time of their admission to the ward. Treatment teams of intervention wards were trained in the SDM-PLUS approach through participation in two half-day workshops. Patients on intervention wards received group training in SDM. Staff (and patients) of the control wards acted under ‘treatment as usual’ conditions. The primary outcome parameter was the patients' perceived involvement in decision-making at 3 weeks after study enrolment, analysed using a random-effects linear regression model.

**Results:**

In total, 161 participants each were recruited in the intervention and control group. SDM-PLUS led to higher perceived involvement in decision-making (primary outcome, analysed patients *n* = 257, mean group difference 16.5, 95% CI 9.0–24.0, *p* = 0.002, adjusted for baseline differences: *β* 17.3, 95% CI 10.8–23.6, *p* = 0.0004). In addition, intervention group patients exhibited better therapeutic alliance, treatment satisfaction and self-rated medication compliance during inpatient stay. There were, however, no significant improvements in adherence and rehospitalisation rates in the 6- and 12-month follow-up.

**Conclusions:**

Despite limitations in patient recruitment, the SDM-PLUS trial has shown that the adoption of behavioural approaches (e.g. motivational interviewing) for SDM may yield a successful application to mental health. The authors recommend strategies to ensure effects are not lost at the interface between in- and outpatient treatment.

Trial registration: The trial was registered at Deutsches Register Klinischer Studien (DRKS00010880).

## Introduction

Psychiatrists and patient representatives increasingly see shared decision-making (SDM) with patients suffering from mental health conditions as an ethical imperative (Drake and Deegan, 2009) which may contribute to greater patient satisfaction and better health outcomes (Beitinger *et al*., 2014). However, there is evidence that SDM is rarely implemented in mental health settings (Hamann *et al*., 2008; McCabe *et al*., 2013), especially in acute inpatient units, where some patients are involuntarily treated (Hamann *et al*., 2016; Giacco *et al*., 2018*a*, *b*). Besides the well-known general barriers to SDM (Legare *et al*., 2008) (e.g. time constraints), specific mental health factors (or factors that may be exacerbated in mental health settings) may be responsible for the underuse of SDM. On the patients' side, these factors include, among others, a lack of interest in decision-making (e.g. due to depressive or negative symptoms) (Hamann *et al*., 2006; Hamann *et al*., 2016), a feeling of powerlessness *vis-a-vis* their providers (Hamann *et al.*, 2016) or passivity in medical consultations (Hamann *et al*., 2014). On the psychiatrists' side, reservations about implementing SDM are often founded in doubts about patients' insight and decisional capacity (Seale *et al*., 2006; Hamann *et al*., 2009; Hamann *et al.*, 2016). Here, psychiatrists might even be afraid to make patient outcomes worse by sharing decision-making and thereby arrive at bad choices.

The aim of the present study was to evaluate a complex intervention for the implementation of SDM (shared decision-making PLUS, i.e. SDM-PLUS (Hamann and Heres, 2014)) that specifically addresses these barriers by expanding SDM techniques with motivational approaches and by intervening on both sides of the clinical encounter, i.e. with both patients and providers in acute treatment settings.

### Aim and hypotheses

The aim of the present study was to evaluate the effects of SDM-PLUS on decision-making patterns on acute psychiatric wards between psychiatrists and patients with schizophrenia. We hypothesised that the intervention would lead to professionals and patients using the skills learned in the SDM-PLUS training, resulting in a higher perceived involvement of patients. We argue that better (perceived) involvement of patients in medical decisions would be a benefit *per se* (Drake and Deegan, 2009). However, a higher perceived involvement from the patients' side may also have an effect on the therapeutic alliance, paving the way for better adherence and fewer relapses caused by non-adherence (Zolnierek and Dimatteo, 2009; Priebe *et al*., 2011).

## Methods

### Trial design, randomisation, blinding

The study was designed as a multi-centre, matched-pair cluster-randomised controlled trial of SDM-PLUS in 12 acute psychiatric wards of five psychiatric state hospitals addressing inpatients with schizophrenia or schizoaffective disease (Hamann *et al*., 2017*a*). SDM-PLUS was implemented on the intervention wards, while on the control wards, treatment as usual (TAU) was continued. The primary investigators (J.H. and S.H.) determined pairs of comparable wards (number of patients, distribution of diagnoses, staff, etc.). For randomisation of wards, a blinded member of our statistical department generated binary random variables using the statistical software R. These binary random variables were then used to assign one of the paired centres to the intervention group and the other to the control group. A cluster-randomised design, in which the unit of randomisation is the psychiatric ward, was seen as necessary to prevent contamination of intervention and control conditions (Craig *et al*., 2008). In order to minimise contamination bias (i.e. staff or patients from control wards becoming familiar with SDM-PLUS), wards were selected to ensure that there was no overlap in personnel and no regular patient transfer between wards.

Due to the nature of the intervention (staff training, patient training), there was no blinding within intervention wards. Likewise, a blinding of raters was not applicable because most ratings, including the main outcome measure, were self-ratings.

### Participants

All patients fulfilling the inclusion criteria were consecutively screened for the trial at the time of their admission to the ward. Inclusion criteria were inpatient status of participating ward, age 18–65 years, diagnosis of schizophrenia or schizoaffective disorder (ICD 10: F20/F25), being capable of participating in 60 min group intervention (according to their clinicians' estimate) and being able to provide written informed consent. Patients were excluded if they suffered from mental retardation and had insufficient proficiency in German to discuss treatment decisions. Involuntary hospitalisation was not an exclusion criterion.

As it became clear quite early during the study course that there would be a considerable number of patients dropping out before the primary outcome, we aimed at compensating for these dropouts by some over-recruitment.

### Intervention and control condition

The SDM-PLUS approach (Hamann and Heres, 2014) was developed by the authors and some parts of it (e.g. patient training) had been evaluated in earlier studies (Hamann *et al*., *2017b*). SDM-PLUS aims to empower health care staff and patients alike with regard to SDM-specific communication techniques. For health care staff, the existing approaches to applying SDM (e.g. Elwyn *et al*., 2017) were expanded to include patients without insight or with reduced decisional capacity. Thus, SDM-PLUS teaches communication techniques derived from motivational interviewing and negotiation approaches (Hamann and Heres, 2014).

The two principal investigators (J.H. and S.H., both clinical psychiatrists) provided interactive workshops on SDM-PLUS techniques to treatment teams (consultants, residents, nurses, psychologists and social workers) working on intervention wards. The two half-day workshops were based on a power point presentation and written case vignettes for role plays and took place in the respective psychiatric hospitals. It was mandatory that all physicians (residents and consultants) of intervention wards and as many members of the nursing team as possible participated in both workshops. In addition to these workshops, physicians were continuously supervised and supported by the study centre in the form of weekly meetings with the physicians in charge. In case of staff changes, one-to-one teaching sessions were offered to new staff members. In the intervention group, all of the participating physicians took part in the staff training. Additionally, 10–50% of the remaining staff (i.e. nurses, psychologists, social workers, occupational therapists) participated in the training.

Patients were provided with group training in SDM (Hamann *et al*., 2011) and the use of question prompt sheets for ward rounds and individual consultations. Throughout the study period, this group training was offered twice a week for all wards and it was ensured that all intervention group patients participated at least in two group sessions.

Staff (and patients) from the control wards acted under TAU conditions but were offered SDM-PLUS training after the end of the study.

### Data obtained

At all time points (baseline, 3 weeks after study enrolment or discharge, whatever happened first, 3, 6, 9 and 12 months after discharge), identical data were collected from the intervention and control groups.

#### Baseline parameters

For all patients recruited, socio-demographics, diagnosis, illness severity (Clinical Global Impression (CGI) and Global Assessment of Functioning (GAF) scores) and data on anamnesis (previous hospitalisations, duration of illness, etc.) were recorded at baseline (at study entry). In addition, we administered the Birchwood Insight Scale (Birchwood *et al*., 1994) and the patients' perception of the current admission using the MacArthur Admission Experience Survey (O'Donoghue *et al*., 2013).

#### Primary outcome

The primary outcome parameter was the patients' perceived involvement in decision-making (regarding drug treatment during the inpatient stay) using the SDM-Q-9 questionnaire at 3 weeks after enrolment in the study or at discharge (whichever occurred first). Following Rodenburg-Vandenbussche *et al*. (2015), we defined a 15-point difference as clinically meaningful.

#### Secondary outcomes at 3 weeks after enrolment

Whether or not the intervention also had an influence on the therapeutic relationship was determined using the Helping Alliance Scale (patient (HAS-P) and a clinician (HAS-C) version) (McCabe *et al*., 2012). Treatment satisfaction was measured using the Questionnaire on Patients' Treatment Satisfaction (ZUF8) and the prevalence of unmet needs on the patients' side after the intervention was assessed using the Camberwell Assessment of Need self-report questionnaire (CANSAS-P). For the assessment of adherence, patients filled out the Medication Adherence Rating Scale (MARS; Thompson *et al*., 2000).

#### Secondary outcomes at 3, 6, 9 and 12 months after discharge

In addition, the aspects of patients' well-being and quality of life were addressed using the WHO-5 well-being index and the EUROHIS-QOL (generic quality of life) (Brähler *et al*., 2007). Finally, information on whether or not there had been any readmissions during the follow-up period was obtained from the patients' outpatient psychiatrists.

### Statistical analyses

The primary analysis was a comparison of SDM-Q-9 sum scores at T1 between the intervention and control groups. For the study to have 80% probability of detecting a mean difference of 15, the study sought to recruit 23 patients in 12 wards each, giving a total of 276 patients. This calculation assumed a two-sided significance level of 5%, a within-cluster standard deviation of 30, intra-cluster coefficient of 0.04, and 20% dropout.

To assess the effect of the intervention on the continuous primary outcome, a mixed-effects linear regression model was fitted with ward (cluster) as a random-effect term and intervention group as a fixed effect. A significance level of *α* = 5% was used. A modified intention-to-treat approach was taken to the analysis, i.e. patients in intervention clusters were analysed in this group (if outcome data were available) even if they did not receive all of the planned intervention. The ICC for the primary outcome variable was estimated using the variance components of the mixed-effects model.

Exploratory analyses were performed to assess the effect of the intervention on the secondary outcome measures. Random-effect linear models were fitted to the data for group comparisons with regard to continuous secondary outcome measures, analogous to the primary analysis. For the binary secondary outcome measure rehospitalisation, a corresponding logistic mixed-effects model was used. To account for baseline differences, we performed sensitivity analyses adjusting for all baseline variables with group differences of *p* < 0.25 (illness severity, diagnosis, admission status and admission experience). As a considerable proportion of patients dropped out before the main outcome could be obtained, we additionally did a sensitivity analysis using multiple imputation methods to predict SDM-Q-9 scores for all patients with missing values.

As all long-term outcomes were non-significant, we decided to report only the most important ones in the text. Results on all outcome measures can be found in the online Supplementary material.

### Ethics, informed consent procedure and trial registration

The trial was approved by the local review board (Ethikkommission der Technischen Universität München). All patients gave written informed consent. The trial was registered at Deutsches Register Klinischer Studien (DRKS00010880) and the study protocol has been published (Hamann *et al.*, 2017*a*).

## Results

**Fig. 1.**
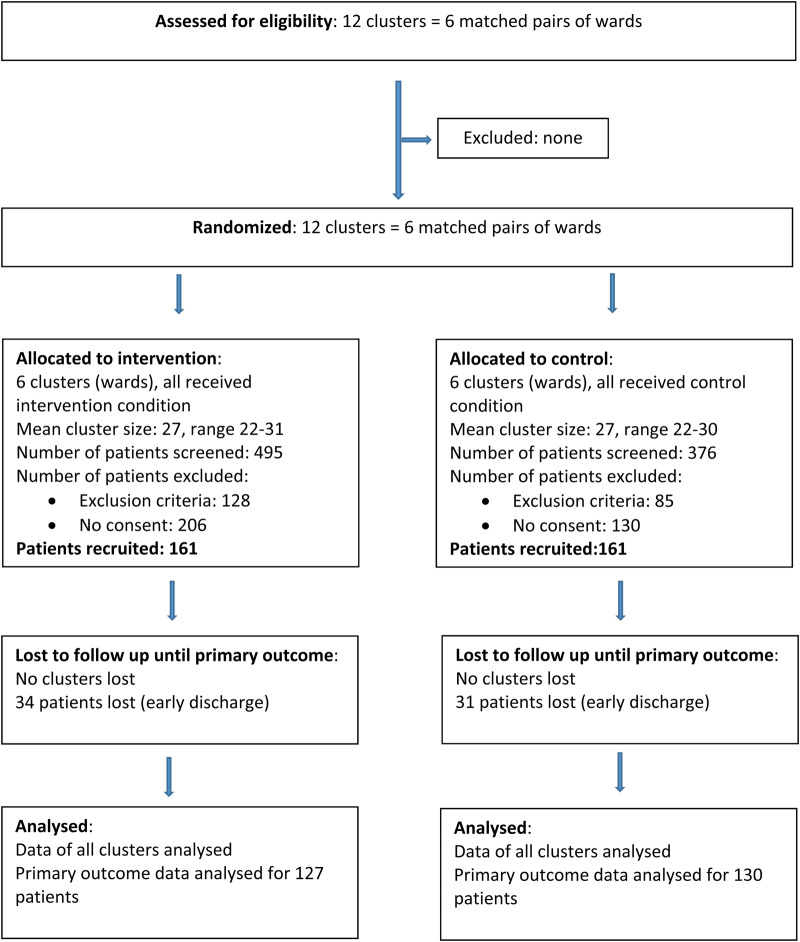
Participant flow (CONSORT diagram).

From October 2016 until March 2018, *N* = 322 inpatients were recruited for the trial. For participant flow, see Fig. 1 (CONSORT diagram). Among patients screened (*n* = 871), most patients were excluded because they did not provide informed consent (i.e. they refused to participate in the trial (*N* = 336; intervention/control 206/130)). In addition, *N* = 94 patients (intervention/control 57/37) were excluded due to insufficient proficiency in German, followed by severe cognitive impairment (*N* = 39; intervention/control 22/17), uncertainty in diagnosed illness (*N* = 31; intervention/control 22/9), age above 65 years (*N* = 20; intervention/control 9/11), severe aggressive behaviour (*N* = 12; intervention/control 9/3). The remaining 18 patients (9 each per group) were excluded for various reasons (e.g. physical disability to communicate, etc.).

### Baseline data

Patients were on average 42 years old and approximately half were female. Most patients were suffering from schizophrenia, and more than a third were admitted involuntarily. Patients had on average a history of illness of 13 years and had had seven previous hospitalisations. According to CGI, they were ‘markedly ill’ at baseline and exhibited a mean GAF score of 37 (see Table 1).

**Table 1.** Baseline data

	Intervention (*N* = 161)	Control (*N* = 161)	*p* value
Age (years, mean, SD)	42.1 (12.9)	41.4 (13.6)	0.61
Gender (female)	84 (52%)	76 (47%)	0.37
Diagnosis	F20: 99 (61%) F25: 52 (32%) Other F2-diagnosis: 10 (6%)	F20: 115 (71%) F25: 38 (24%) Other F2-diagnosis: 8 (5%)	0.21
Involuntary admissions	61 (40%)	41 (28%)	0.03
Legal guardianship	94 (61%)	86 (58%)	0.31
Duration of illness (years, mean, SD)	12.4 (10.3)	13.0 (11.3)	0.62
Previous hospital stays (mean, SD)	6.9 (6.7)	7.6 (7.5)	0.39
CGI (mean, SD)	5.3 (0.9)	5.5 (0.8)	0.04
GAF (mean, SD)	37.7 (13.3)	36.1 (12.4)	0.28
Insight (mean score, SD)	7.7 (2.9)	8.0 (3.1)	0.45
MacArthur Admission Experience (mean score, SD)	2.5 (2.0)	2.1 (1.9)	0.13

### Primary outcome

The intervention yielded a significant (*p* = 0.002) effect on the primary outcome with a mean group difference of 16.5 points in the SDM-Q-9 sum score (95% confidence interval 9.0–24.0, *T*-statistics 4.3, ICC = 0.02), indicating that the intervention group felt more involved in decision-making (regarding antipsychotic medication) than the control group. In the sensitivity analyses, this result remained stable when accounting for baseline differences (*β* 17.3, 95% confidence interval 10.8–23.8, *T*-statistics 5.2, *p* = 0.0004) and when using multiple imputation methods (*β* 13.4, *T*-statistics 3.7, 95% confidence interval 6.2–20.6, *p* = 0.007).

### Secondary outcomes

The positive finding regarding the primary outcome (perceived involvement in decision-making) is also mirrored in group differences regarding therapeutic alliance, treatment satisfaction and self-reported adherence (drug attitudes). Thus, patients in the intervention group had a better alliance, were more satisfied and had better drug attitudes/self-rated adherence than patient in the control group at 3 weeks after study entry (Table 2).

**Table 2.** Secondary outcomes at 3 weeks after enrolment

	Unadjusted model					Adjusted model				
	*β*	CI lower	CI upper	*t*	*p* value	*β*	CI lower	CI upper	*t*	*p* value
Helping Alliance Scale (HAS-P) *N* = 257	1.09	0.31	1.88	2.75	0.02	1.07	0.39	1.74	3.11	0.01
Helping Alliance Scale (HAS-C) *N* = 298	−0.37	−0.97	0.24	−1.18	0.27	−0.42	−0.94	0.11	−1.54	0.15
Treatment satisfaction (ZUF8) *N* = 257	2.73	0.74	4.72	2.69	0.02	3.04	1.69	4.39	4.42	0.001
Camberwell Assessment of Need self-report questionnaire (CANSAS-P) (Number of items with unmet needs) *N* = 256	−0.71	−1.88	0.46	−1.19	0.26	−0.79	−1.91	0.33	−1.38	0.20
Medication Adherence Rating Scale (MARS) *N* = 256	1.17	0.41	1.93	3.02	0.01	1.22	0.59	1.86	3.77	0.004
Clinical Global Impression (CGI) *N* = 308	−0.29	−0.88	0.30	−0.97	0.36	−0.23	−0.71	0.24	−0.96	0.36
Global Assessment of Functioning (GAF) *N* = 308	4.72	−1.76	11.20	1.43	0.18	4.09	−0.79	8.97	1.65	0.13
Duration of inpatient stay (days) *N* = 137	8.48	−16.83	33.78	0.66	0.53	11.78	−12.17	35.72	0.96	0.36

There were, however, no group differences regarding the other outcome measures including CGI, GAF and number of unmet needs. Patients in the two groups also did not differ with regard to the duration of inpatient stay.

### Secondary long-term outcomes

During follow-up, patients and their outpatient psychiatrists were surveyed with regard to adherence, quality of life and readmissions. Overall, there were no significant group differences on these dimensions (Table 3).

**Table 3.** Secondary outcomes at 6 and 12 months follow-up

	Unadjusted model					Adjusted model				
	*β*	CI lower	CI upper	*t*	*p* value	*β*	CI lower	CI upper	*t*	*p* value
Medication Adherence Rating Scale (MARS) 6 months, *N* = 82	0.51	−0.52	1.55	0.97	0.35	0.32	−0.82	1.45	0.55	0.60
Medication Adherence Rating Scale (MARS) 12 months, *N* = 14	−0.71	−3.03	1.61	−0.60	0.58	0.26	−1.97	2.49	0.23	0.83
Well-being (WHO5) 6 months, *N* = 83	3.80	−8.97	16.57	0.58	0.57	3.96	−10.32	18.23	0.54	0.60
Quality of Life (EurohisQuol) 6 months, *N* = 83	1.99	−0.99	4.97	1.31	0.22	1.59	−1.47	4.64	1.02	0.33
	OR	CI lower	CI upper	*z*	*p* value	OR	CI lower	CI upper	*z*	*p* value
Rehospitalisation within 12 months 42/113 (37%) (intervention) 36/116 (31%)(control), *N* = 229	1.31	0.76	2.27	0.98	0.33	1.39	0.78	2.47	1.13	0.26

## Discussion

To date, several studies have evaluated interventions that promote SDM in mental health settings. However, few have addressed very acutely ill patients or those being treated involuntarily (Giacco *et al.*, 2018*b*) and most have had negative results or shown only modest effects.

The present SDM-PLUS approach for patients with schizophrenia or schizoaffective disorder in very acute mental health inpatient settings indicates that a complex intervention which addresses both patients and health care staff changes patients' perception of therapeutic decision-making during an inpatient stay. This was true for the primary outcome (patients' perceived involvement in decision-making), as well as for therapeutic alliance, treatment satisfaction and self-rated medication compliance. There were, however, no long-term changes in the period after discharge.

### Limitations

During the study period, consecutive recruitment was adopted to avoid recruitment bias. However, more patients in the intervention group declined to participate in the trial, which might have biased our results. However, when accounting for baseline differences (e.g. in the intervention group, more patients were admitted involuntarily), group differences (primary and secondary outcomes) remained stable, indicating that the potential influence of a recruitment bias is limited.

In addition, the extent to which the proposed strategies were actually implemented by health care staff might be a potential methodological limitation, especially as we were not able to observe a standardised conversation between patients and their therapists.

Finally, the number of questionnaires returned by patients during follow-up was rather low, weakening the validity of some of our secondary outcomes.

### Discussion of results

The specific feature of SDM-PLUS is the adoption of the SDM approach for the more severely ill patients in mental health, thus, to those patients who might refuse treatment due to a lack of insight, who might be treated involuntarily or who might just be too ill in the view of their psychiatrists to reasonably engage in SDM (Hamann *et al.*, 2009).

The major aim of this trial was to show that SDM-PLUS can lead to more SDM in this group of patients, as reflected in a higher perceived involvement of these patients in medical decision-making. While one might argue that involvement of patients alone might not constitute a clinical or even financial benefit, we claim that the proof of enhanced involvement through the introduction of the SDM-PLUS approach has at least two important implications.

First, users have clear expectations towards the mental health system (Bramesfeld *et al*., 2007) that include aspects such as dignity and autonomy. The implementation of SDM-PLUS thus may help implementing these important aspects. Second, our results show that being highly symptomatic does not prevent patients from sharing decisions. Neither, patients' symptoms should prevent psychiatrists from supporting patient autonomy. Based on our results and other reports (Burn *et al*., 2019), the argument can therefore be reasonably made that even in the very acute mental health settings, the desire of many patients to be addressed as competent individuals (as is in any case ethically required) is possible and can be implemented.

Our trial has shown that the SDM-PLUS intervention not only achieved its primary aim but also that important secondary outcomes during inpatient stay were positively influenced in the same direction. Thus, treatment satisfaction, therapeutic alliance and self-reported adherence were also improved in the intervention group. This is in line with findings that interventions for involuntarily treated inpatients, such as structured patient-centred care planning, have been shown to improve long-term outcomes (Giacco *et al*., 2018*a*, *b*).

These positive results were not continued during follow-up after discharge from the hospital. While improvements during inpatient treatment are positive achievements *per se*, the fact that the effects of the intervention diminished after discharge should be discussed. One major reason might be that health care staff in outpatient treatment (i.e. any psychiatrists practising in the region) was not trained in the SDM-PLUS approach. Further, patient training alone did not show effects on alliance and adherence (Hamann *et al.*, 2017*b*). Finally, the number of observed cases during follow-up was rather low.

### Implications

We believe that the results of the SDM-PLUS trial have several important implications:

First, an expansion of SDM to the more severely ill patients in mental health is possible. This is good news for all patients being treated on closed wards, because it invalidates general excuses for not engaging these patients in medical decision-making. Second, expansions of SDM may need to adopt behavioural approaches (such as motivational interviewing) to be successful with some patients. In addition, training patients may also be necessary to create a ward atmosphere congenial to SDM. Third, the SDM approach appears to change important patterns during inpatient treatment. However, while inpatients transit to outpatient treatment (and thereby change health care staff), positive effects are lost. Strategies for ensuring a continuation of SDM measures at the interface between in- and outpatient treatment need to be developed.

Finally, the SDM-PLUS approach (as all previous SDM approaches) does not yet account for the important role (informal) caregivers may play for the long-term course of mental illnesses. Studies addressing the prospects and modes of caregiver involvement in SDM (Hamann and Heres, 2019) are therefore of great interest, as their ‘triadic’ inclusion may boost the effects of SDM.

## Supplementary material

For supplementary material accompanying this paper visit http://dx.doi.org/10.1017/S2045796020000505. click here to view supplementary material

## Data

The datasets generated during and/or analysed during the current study are available from the corresponding author on reasonable request.
