# Supplementary material for: Implementing shared decision-making on acute psychiatric wards: a cluster-randomized trial with inpatients suffering from schizophrenia (SDM-PLUS)
Source: Epidemiol Psychiatr Sci. 2020 Jun 16;29:e137. doi: 10.1017/S2045796020000505 (PMC7303792; doi:10.1017/S2045796020000505)
Supplement: Supplementary file 1 [file S2045796020000505sup001.docx]

|  | **Unadjusted** | | | | | **Adjusted** | | | | |
| --- | --- | --- | --- | --- | --- | --- | --- | --- | --- | --- |
|  | **beta** | **CI lower** | **CI upper** | **t** | **p value** | **beta** | **CI lower** | **CI upper** | **t** | **p value** |
| SDMQ9 T1 | 16.450 | 8.963 | 23.937 | 4.307 | 0.00155 | 17.302 | 10.772 | 23.832 | 5.193 | 0.00041 |
| SDMQ9 T2 | 4.111 | -8.050 | 16.272 | 0.663 | 0.52257 | 4.624 | -8.360 | 17.608 | 0.698 | 0.50112 |
| SDMQ9 T3 | 0.694 | -10.549 | 11.938 | 0.121 | 0.90606 | 3.107 | -8.872 | 15.085 | 0.508 | 0.62227 |
| SDMQ9 T4 | -1.127 | -21.599 | 19.345 | -0.108 | 0.91710 | 6.063 | -11.438 | 23.565 | 0.679 | 0.52242 |
| API T0 | 0.272 | -0.946 | 1.490 | 0.438 | 0.67072 | 0.009 | -0.969 | 0.987 | 0.017 | 0.98658 |
| API T1 | -0.011 | -1.459 | 1.436 | -0.015 | 0.98795 | -0.199 | -1.390 | 0.992 | -0.328 | 0.75001 |
| API T2 | 0.658 | -0.920 | 2.236 | 0.817 | 0.43292 | 0.658 | -1.137 | 2.453 | 0.718 | 0.48899 |
| API T3 | 0.477 | -1.394 | 2.347 | 0.499 | 0.62832 | 0.569 | -1.486 | 2.624 | 0.543 | 0.59928 |
| API T4 | -0.836 | -3.338 | 1.666 | -0.655 | 0.52883 | -0.175 | -2.673 | 2.323 | -0.138 | 0.89400 |
| API T5 | 1.304 | -3.185 | 5.793 | 0.569 | 0.59386 | -0.358 | -4.463 | 3.747 | -0.171 | 0.87099 |
| PatPart19 T0 | 1.571 | -1.210 | 4.352 | 1.107 | 0.29423 | 1.099 | -1.491 | 3.688 | 0.832 | 0.42503 |
| PatPart19 T1 | 3.020 | 0.326 | 5.714 | 2.197 | 0.05269 | 2.884 | 0.103 | 5.665 | 2.032 | 0.06954 |
| MARS T1 | 1.168 | 0.409 | 1.927 | 3.017 | 0.01297 | 1.222 | 0.586 | 1.858 | 3.765 | 0.00369 |
| MARS T2 | -0.011 | -1.092 | 1.069 | -0.021 | 0.98377 | 0.155 | -1.011 | 1.321 | 0.260 | 0.79985 |
| MARS T3 | 0.513 | -0.523 | 1.550 | 0.971 | 0.35432 | 0.317 | -0.815 | 1.449 | 0.548 | 0.59558 |
| MARS T4 | -0.182 | -1.840 | 1.477 | -0.215 | 0.83466 | -0.442 | -2.348 | 1.465 | -0.454 | 0.66186 |
| MARS T5 | -0.708 | -3.030 | 1.613 | -0.598 | 0.58200 | 0.264 | -1.966 | 2.494 | 0.232 | 0.82806 |
| HASP T1 | 1.094 | 0.313 | 1.875 | 2.746 | 0.02063 | 1.065 | 0.394 | 1.736 | 3.111 | 0.01104 |
| HASP T2 | 0.457 | -0.521 | 1.435 | 0.916 | 0.38111 | 0.488 | -0.603 | 1.579 | 0.876 | 0.40136 |
| HASP T3 | 0.410 | -0.442 | 1.262 | 0.943 | 0.36814 | 0.326 | -0.595 | 1.247 | 0.693 | 0.50390 |
| HASP T4 | 1.497 | 0.117 | 2.876 | 2.126 | 0.06239 | 1.753 | 0.258 | 3.249 | 2.299 | 0.05058 |
| HASP T5 | 0.078 | -2.053 | 2.209 | 0.071 | 0.94589 | 0.374 | -2.102 | 2.850 | 0.296 | 0.77896 |
| ZUF8 T1 | 2.727 | 0.739 | 4.715 | 2.688 | 0.02277 | 3.040 | 1.693 | 4.387 | 4.424 | 0.00129 |
| WHO5 T3 | 3.799 | -8.972 | 16.569 | 0.583 | 0.57279 | 3.958 | -10.315 | 18.231 | 0.544 | 0.59866 |
| WHO5 T5 | 6.929 | -17.410 | 31.267 | 0.558 | 0.60093 | 4.952 | -29.815 | 39.720 | 0.279 | 0.79129 |
| EUROHIS T3 | 1.987 | -0.992 | 4.966 | 1.307 | 0.22035 | 1.586 | -1.468 | 4.641 | 1.018 | 0.33262 |
| EUROHIS T5 | 1.179 | -4.386 | 6.743 | 0.415 | 0.69527 | 1.801 | -5.598 | 9.201 | 0.477 | 0.65340 |
| CGI T1 | -0.291 | -0.883 | 0.300 | -0.965 | 0.35730 | -0.233 | -0.710 | 0.243 | -0.959 | 0.36000 |
| CGI T2 | 0.051 | -0.282 | 0.385 | 0.302 | 0.76913 | 0.043 | -0.317 | 0.402 | 0.233 | 0.82022 |
| CGI T3 | -0.186 | -0.832 | 0.459 | -0.565 | 0.58445 | -0.091 | -0.753 | 0.572 | -0.268 | 0.79467 |
| CGI T4 | 0.064 | -0.573 | 0.701 | 0.198 | 0.84709 | 0.199 | -0.455 | 0.853 | 0.597 | 0.56378 |
| CGI T5 | -0.019 | -0.823 | 0.784 | -0.047 | 0.96396 | 0.052 | -1.111 | 1.216 | 0.088 | 0.93316 |
| GAF T0 | 1.216 | -5.139 | 7.571 | 0.375 | 0.71548 | -0.320 | -2.649 | 2.009 | -0.269 | 0.79303 |
| GAF T1 | 4.718 | -1.760 | 11.196 | 1.428 | 0.18389 | 4.092 | -0.785 | 8.969 | 1.645 | 0.13107 |
| GAF T2 | -0.988 | -6.365 | 4.390 | -0.360 | 0.72641 | -1.106 | -7.152 | 4.940 | -0.359 | 0.72738 |
| GAF T3 | -2.616 | -12.124 | 6.892 | -0.539 | 0.60146 | -3.802 | -12.902 | 5.298 | -0.819 | 0.43398 |
| GAF T4 | 0.189 | -10.130 | 10.508 | 0.036 | 0.97212 | -1.852 | -12.667 | 8.963 | -0.336 | 0.74408 |
| GAF T5 | 3.221 | -7.562 | 14.004 | 0.585 | 0.58368 | -0.546 | -12.321 | 11.229 | -0.091 | 0.93107 |
| HASC T1 | -0.366 | -0.974 | 0.243 | -1.179 | 0.26586 | -0.416 | -0.943 | 0.112 | -1.544 | 0.15369 |
| HASC T2 | 0.367 | -0.438 | 1.172 | 0.894 | 0.39223 | 0.303 | -0.524 | 1.130 | 0.719 | 0.48887 |
| HASC T3 | 0.057 | -0.633 | 0.746 | 0.162 | 0.87467 | -0.025 | -0.797 | 0.747 | -0.064 | 0.95045 |
| HASC T4 | -0.178 | -1.281 | 0.924 | -0.317 | 0.75761 | -0.312 | -1.554 | 0.930 | -0.492 | 0.63329 |
| HASC T5 | -0.130 | -1.389 | 1.128 | -0.203 | 0.84744 | 0.235 | -1.179 | 1.649 | 0.326 | 0.75796 |
| SDMQD T1 | 1.416 | -4.520 | 7.352 | 0.468 | 0.65007 | 1.604 | -4.682 | 7.891 | 0.500 | 0.62774 |
| SDMQD T2 | 4.352 | -3.073 | 11.776 | 1.149 | 0.27736 | 4.607 | -3.246 | 12.460 | 1.150 | 0.27693 |
| SDMQD T3 | 4.500 | -1.808 | 10.808 | 1.398 | 0.19229 | 3.659 | -3.865 | 11.182 | 0.953 | 0.36543 |
| SDMQD T4 | -2.913 | -13.783 | 7.958 | -0.525 | 0.61092 | -5.940 | -17.974 | 6.094 | -0.967 | 0.35613 |
| SDMQD T5 | 3.047 | -9.892 | 15.986 | 0.462 | 0.66377 | 6.880 | -14.726 | 28.486 | 0.624 | 0.55992 |
| CANSAS_T1 | -0.709 | -1.876 | 0.458 | -1.190 | 0.26139 | -0.788 | -1.910 | 0.333 | -1.377 | 0.19841 |
